# PPP2R5D promotes hepatitis C virus infection by binding to viral NS5B and enhancing viral RNA replication

**DOI:** 10.1186/s12985-022-01848-5

**Published:** 2022-07-14

**Authors:** Muhammad Ikram Anwar, Ni Li, Qing Zhou, Mingxiao Chen, Chengguang Hu, Tao Wu, Haihang Chen, Yi-Ping Li, Yuanping Zhou

**Affiliations:** 1 Guangdong Provincial Key Laboratory of Gastroenterology, Department of Gastroenterology and Hepatology Unit, Nanfang Hospital, Southern Medical University, Guangzhou, China; 2 Institute of Human Virology, Zhongshan School of Medicine, and Key Laboratory of Tropical Disease Control of Ministry of Education, Sun Yat-Sen University, Guangzhou, 510080 China; 3 Department of Infectious Diseases, Hainan General Hospital, Hainan Affiliated Hospital of Hainan Medical University, Haikou, China; 4 Department of Infectious Diseases, The Fifth Hospital of Sun Yat-Sen University, Zhuhai, China

**Keywords:** Hepatitis C virus, PPP2R5D, Protein–protein interaction, RNA dependent RNA polymerase, Phosphatase activity

## Abstract

**Background:**

Hepatitis C virus (HCV) infection increased the risk of hepatocellular carcinoma. Identification of host factors required for HCV infection will help to unveil the HCV pathogenesis. Adaptive mutations that enable the replication of HCV infectious clones could provide hints that the mutation-carrying viral protein may specifically interact with some cellular factors essential for the HCV life cycle. Previously, we identified D559G mutation in HCV NS5B (RNA dependent RNA polymerase) important for replication of different genotype clones. Here, we searched for the factors that potentially interacted with NS5B and investigated its roles in HCV infection.

**Methods:**

Wild-type-NS5B and D559G-NS5B of HCV genotype 2a clone, J6cc, were ectopically expressed in hepatoma Huh7.5 cells, and NS5B-binding proteins were pulled down and identified by mass spectrometry. The necessity and mode of action of the selected cellular protein for HCV infection were explored by experiments including gene knockout or knockdown, complementation, co-immunoprecipitation (Co-IP), colocalization, virus infection and replication, and enzymatic activity, etc.

**Results:**

Mass spectrometry identified a number of cellular proteins, of which protein phosphatase 2 regulatory subunit B’delta (PPP2R5D, the PP2A regulatory B subunit) was one of D559G-NS5B-pulled down proteins and selected for further investigation. Co-IP confirmed that PPP2R5D specifically interacted with HCV NS5B but not HCV Core and NS3 proteins, and D559G slightly enhanced the interaction. NS5B also colocalized with PPP2R5D in the endoplasmic reticulum. Knockdown and knockout of PPP2R5D decreased and abrogated HCV infection in Huh7.5 cells, respectively, while transient and stable expression of PPP2R5D in PPP2R5D-knockout cells restored HCV infection to a level close to that in wild-type Huh7.5 cells. Replicon assay revealed that PPP2R5D promoted HCV replication, but the phosphatase activity and catalytic subunit of PP2A were not affected by NS5B.

**Conclusions:**

PPP2R5D interactes with HCV NS5B and is required for HCV infection in cultured hepatoma cells through facilitating HCV replication.

## Introduction

The majority of hepatitis C virus (HCV) infecting patients develop chronic hepatitis C, which is associated with increasing risk of fibrosis, cirrhosis, and hepatocellular carcinoma (HCC). To date, ~ 71 million people are chronically infected by HCV, with ~ 1.75 million new infections and ~ 0.7 million deaths annually [1]. In recent years, treatment of hepatitis C has been revolutionarily improved by the implement of interferon-free direct-acting antivirals (DAAs) regimens, which significantly increases the cure rate above 95%. Although great success has been achieved for HCV therapy, challenges remain, such as lack of an HCV vaccine, low access to DAA therapy in some countries and regions, HCC and reinfection after the cure, emergence of drug resistance, etc. The pathogenesis of HCV infection is also incompletely understood.

HCV is a member of the *Hepacivirus* genus in the *Flaviviride* family. The positive RNA genome of 9.6 kb encodes a single polyprotein of ~ 3,010 amino acids, flanked by 5’ and 3’ untranslated regions (UTRs), necessary for viral RNA replication, translation, and stability [2]. The 5’UTR contains a highly structured internal ribosomal entry site (IRES), which helps to initiate polyprotein translation. The polyprotein is cleaved by both host and viral proteases to produce three structural proteins (core, E1 and E2) and seven non-structural proteins (p7, NS2, NS3, NS4A, NS4B, NS5A and NS5B). Structural proteins form HCV virions, while non-structural proteins are primarily responsible for viral RNA replication, translation, polyprotein processing, virus assembly, and release [2].

HCV NS5B is an RNA dependent RNA polymerase required for viral RNA replication [2]. Numerous host factors have been identified to be involved in and regulate HCV RNA replication by different mechanisms, such as acting on viral proteins, RNA, or the replication complex [3, 4]. In general, virus propagation in the cell displays a complex landscape of virus-host interaction and uncovering the host factors involved is critical for understanding the mechanism of HCV RNA replication and viral pathogenesis.

In the development of infectious HCV recombinants and full-length clones, cell culture adaptive mutations have been identified and demonstrated to enable or enhance RNA replication or virus production by accelerating the complete HCV life cycle [5, 6]. These mutations may have changed the virus-host interaction to be beneficial for HCV replication in the cell, thus providing clues that cellular factors important for HCV infection could be identified though its interaction with the viral protein carrying adaptive mutations. We previously identified three key mutations, F1464L/A1672S/D2979G (LSG), that permitted the replication of full-length HCV clones of genotype 2a and 2b [5–7] and subsequenctly genotypes 1a, 2c, 3a and 6a [5, 6, 8–10]. These studies have demonstrated that the LSG mutations enhanced HCV RNA replication and virus production. However, the underlying mechanism of LSG function is still elusive. The “G” mutation D2979G is located in NS5B thumb domain, corresponding to D559G residue of NS5B protein (D559G-NS5B). The D559G change has been predicted to reduce the efficacy of HCV antivirals in a bioinformatics study [11]. In this study, we set out to identify D559G-NS5B-binding proteins and study the role of selected protein in HCV infection. We found that PPP2R5D was pulled down by D559G-NS5B and specifically interacted with NS5B, which led to further demonstration that PPP2R5D was required for HCV infection and could enhance HCV replication.

## Materials and Methods

### Plasmids and Reagents

Plasmid expressing D559G-NS5B was amplified from J6cc clone [6], and wild-type NS5B (WT-NS5B) was made by changing G to D at amino acid 559 by PCR. The N-terminus of NS5B was fused with Flag-tag (NH_2_-DYKDDDDK-COOH), and Flag-NS5B was cloned into the vector plasmid pcDNA3.1 (Addgene, USA). HA-tagged HCV Core and NS3 were also constructed. PPP2R5D gene was amplified from human hepatoma cell line Huh7.5 cells (generously provided by Dr. Charles Rice, Apath L.L.C. and Rockefeller University, USA), and HA-tag (NH_2_-YPYDVPDYA-COOH) was added at either N- or C-terminus of PPP2R5D by fusion PCR procedure, cloned into the pEGFP-C1 (Addgene) replacing the EGFP sequence, and designated HA/n-PPP2R5D or PPP2R5D-HA/c, respectively. Untagged or Flag-tagged PPP2R5D was also constructed as required. All of the constructs were confirmed by DNA sequencing (Sangon Biotech Company, China). Endoplasmic reticulum (ER) plasmid DsRed2-ER-5 (kindly provided by Dr. Liangyi Chen, Peking University, China).

The antibodies used for western blotting included mouse monoclonal antibodies, anti-Core (ab2740; Abcam, UK), anti-NS3 (ab65407; Abcam), anti-Flag M2 (F3165; Sigma-Aldrich), anti-Flag (PM020, MBL, Japan), anti-HA (sc-7392; Santa Cruz, USA or M180-3, MBL), anti-PP2A C subunit (52F8) (both alpha and beta isoforms) Rabbit mAb (P67775, Cell Signaling Technology, USA), and anti-β-actin (ab8224; Abcam). The secondary antibodies were goat anti-mouse conjugated with horseradish peroxidase (ProteinTech, China) or with Alexa Fluor 488, 594, and 647, and anti-goat Alexa Fluor 568 (Life Technologies, China). ECL™ anti-mouse IgG and an HRP-linked whole antibody (GE Healthcare, UK) were used for the focus forming unit (FFU) assay. Chemicals 4′,6-diamidino-2-phenylindole (DAPI) (Thermo Fisher Scientific, USA) and serine/threonine phosphatase substrate 6,8-difluoro-4-methyl-umbelliferyl phosphate (DiFMUP) (No. 11627, AAT Bioquest, CA, USA) was purchased.

### Cell culture and transfection

Huh7.5 cells and HEK293T cells were maintained in Dulbecco’s modified Eagle’s medium (DMEM) (Gibco, Thermo Fisher Scientific) supplemented with 10% fetal bovine serum at 37 °C with 5% CO_2_. Transfections of HEK293T or Huh7.5 cells were performed by use of Lipofectamine 2000 (Invitrogen, USA) as per the manufacturer's instructions.

### HCV RNA transfection, virus production, and focus forming unit (FFU) assay

HCV RNA transfection, virus production and focus forming unit (FFU) assay were performed as previously described. [5, 6, 12]. Briefly, FFU assay was performed as the followings: 6 × 10^3^ Huh7.5 cells were seeded in polylysine-coated 96-well plates for 24 h, and then infected with serial dilutions of HCV for 48 h. The cells were fixed and immunostained with the anti-HCV Core antibody C7-50 (Santa Cruz Biotechnology, USA), and the percentage of HCV Core positive cells was enumerated under a fluorescence microscope.

### HCV infection

Huh7.5 cells with wild-type (WT) and PPP2R5D-knockout (KO) Huh7.5 cells were seeded in 6-well plates (3 × 10^5^ cells per well) and allowed to grow for ~ 16 h. Then, the cells were transfected with PPP2R5D and left for 16 h, followed by HCV infection (multiplicity of infection [MOI] of 0.01) for 48 h. The cells were lysed, and HCV Core protein was detected by western blotting. Total RNA was extracted from infected cells, and the level of HCV RNA was determined by qRT-PCR.

### Generation of PPP2R5D knockdown and knockout (KO) cell lines

To generate PPP2R5D knockdown cells, lentivirus pLKO.1 vector (Addgene) was used to express PPP2R5D-targeting short hairpin RNA (shRNA). Two sets of shRNAs were designed, PPP2R5D-sh-F1 (5’-CCGGAGTCTGACTGAGCCGGTAATTCTCGAGAATTACCGGCTCAGTCAGACTTTTTTG-3’) and PPP2R5D-sh-R1 (5’-AATTCAAAAAAGTCTGACTGAGCCGGTAATTCTCGAGAATTACCGGCTCAGTCAGACT-3’), PPP2R5D-sh-F2 (5’-CCGGCTTGCTCTCCTAGACCTATTTCTCGAGAAATAGGTCTAGGAGAGCAAGTTTTTG-3’) and PPP2R5D-sh-R2 (5’-AATTCAAAAACTTGCTCTCCTAGACCTATTTCTCGAGAAATAGGTCTAGGAGAGCAAG-3’); scramble control shRNAs were also used. PPP2R5D-knockdown Huh7.5 cells were generated by lentivirus infection and puromycin (2 μg$\mu$/ml) selection. Small interfering RNAs (siRNAs) targeting PPP2R5D gene were synthesized and used for the silencing experiment of Huh7.5 cells harboring HCV JFH1 subgenomic replicon (genotype 2a). The siRNAs were siPPP2R5D-1 sense (5’-GAGCCUGAUAAGUGACAAUTT-3’) and antisense (5’-AUUGUCACUUAUCAGGCUCTT-3’); siPPP2R5D-2 sense (5’-GCCACUGGAACAAGACAAUTT-3’) and antisense (5’-AUUGUCUUGUUCCAGUGGCTT-3’); siPPP2R5D-3 sense (5’-GGCAUACUGUGUGGUACAATT-3’) and antisense (5’-UUGUACCACACAGUAUGCCTT-3’); siRNA negative controls (siNC) sense (5’-ACGUGACACGUUCGGAGAATT-3’) and antisense (5’-ACGUGACACGUUCGGAGAATT-3’). Knockout (KO) of Huh7.5 PPP2R5D was made by CRISPR-Cas9 technology using sgRNAs, PPP2R5D-1 (5’-CACCGGCTCCGGGCTTATATCCGT-3’ and 5’-AAACACGGATATAAGCCCGGAGCC-3’) and PPP2R5D-2 (5’-CACCTAGCCGTGATGTTGTCACTG-3’, and 5’-AAACCAGTGACAACATCACGGCTA-3’). Control scramble sgRNAs were 5’-CACCGACGGAGGCTAAGCGTCGCAA-3’ and 5’-AAACTTGCGACGCTTAGCCTCCGTC-3’. Synthetic double-stranded DNA fragments were cloned in pSpCas9 BB-2A-Puro PX459 (Addgene) [13]. The PPP2R5D-KO cells were maintained in the presence of puromycin (1 µg/mL) and replenished every 2–3 days. The disruption of PPP2R5D in Huh7.5 KO cell lines was confirmed by sequencing.

To select a monoclonal PPP2R5D-KO cell line that stably expressed PPP2R5D (designated PPP2R5D-Compl), untagged PPP2R5D expressing plasmid were transfected into PPP2R5D-KO cells. After puromycin selection (2 µg/mL) for 72 h, the cells were diluted into 96-well plates, allowed to expand, and identified the cell clones expressing PPP2R5D by western blotting.

### Pull-down assay and co-immunoprecipitation

Pull-down assay was performed to identify the proteins that potentially interact with HCV NS5B. Briefly, Flag-tagged WT-NS5B or D559G-NS5B (N-terminus, n-Flag) were constructed and transfected into Huh7.5 cells. The cells were lysed and incubated with the anti-FLAG M2 antibody for 2 h. Then, the Flag-tagged NS5B was pulled-down using M2-conjugated agarose beads and eluted from the beads. Pulled-down proteins was subjected to SDS-PAGE, silver staining, and mass spectrometry analysis.

For co-immunoprecipitation (Co-IP) experiment, 293 T cells were co-transfected with Flag-tagged D559G-NS5B or WT-NS5B together with HA-tagged PPP2R5D using Lipofectamine 2000 reagent (Life Technologies). The transfected cells were lysed with 500 μl of IP lysis buffer [50 mM Tris–HCl (pH = 7.4), 1% NP-40, 0.25% Na-deoxycholate, 150 mM NaCl, 1 mM EDTA, 1 mM Na_3_VO_4_, 1 mM NaF, and 1% cocktail protein inhibitors], incubated on ice for 60 min, and clarified by centrifugation at 12,000 g at 4 °C for 10 min. The concentration of total protein was determined by the BCA Protein Assay kit (GenStar, China). For each Co-IP, the cell lysates of 450 μl were incubated with 5 μg of antibody under gentle rotation at 4 °C for 2 h, then 25 μl of protein A-agarose beads (Santa Cruz Biotechnology) was added and left with gentle rotation at 4 °C overnight. The beads were then washed five times in IP lysis buffer, and the bound proteins were eluted and analyzed by SDS-PAGE and western blotting.

### Western blotting

Total protein was separated by SDS-PAGE (10% PAGE, 60–100 μg/lane), and then transferred to a PVDF membrane (0.2 μm) (Bio-Rad, USA). The transferred membrane was blocked with 5% milk at room temperature for 1 h and incubated with primary antibodies at 4 °C overnight. A secondary antibody was applied at room temperature for 2 h. Protein bands were visualized with an ECL chemiluminescence kit (Proteintech, China) and OPTIMAX X-Ray Film Processor (PROTEC GmbH, Germany).

### qRT-PCR

Total RNAs were extracted and quantified using Nanodrop-2000 (Thermo Fisher Scientific), and cDNA was synthesized from 1 µg of RNA using SuperScript VILO cDNA Synthesis Kit (Thermo Fisher Scientific). Real-time PCR was performed using TaqMan Universal master mix II (4440038, Applied Biosystems, USA) or an ABI 7500 real-time PCR system (Applied Biosystems). GAPDH was used as internal control. For quantification of HCV RNA, qRT-PCR was performed using primers targeting HCV 5’UTR (Fwd-HCV, 5’-GCAGAAAGCGTCTAGCCAT-3’ and Rev-HCV, 5’-CTCGCAAGCACCCTATCAG-3’). Each reaction was performed in triplicate, and the levels of RNAs were calculated by the comparative threshold cycle (C_T_) method (ΔΔC_T_) and normalized to the level of GAPDH. The expression pattern was also analyzed by western blotting using the anti-HCV Core antibody.

### Detection of phosphatase activity

To detect the phosphatase activity, a serine/threonine phosphatase substrate 6,8-difluoro-4-methyl-umbelliferyl phosphate (DiFMUP) was used (AAT Bioquest, CA, USA). Briefly, total protein level of cell lysates was determined by BCA Protein Assay kit (GenStar, China). A volume of 48 μl of cell lysates mixed with 2 μl NiCl_2_ (40 mM) were added to 50 μl 1× reaction buffer (50 mM Tris [pH 7.5] and 150 mM NaCl and 1 mM EDTA) containing reconstituted DiFMUP. After incubation at 37 °C for 30 min, plates were subjected to fluorescence measurement at excitation and emission wavelengths of 355 nm and 460 nm, respectively.

### Confocal microscopy

Colocalization of NS5B and PPP2R5D was detected by confocal microscopy. Huh7.5 cells were transfected with Flag-NS5B and HA-PPP2R5D for 36 h. The cells were fixed with 4% paraformaldehyde in PBS at room temperature for 15 min, washed by PBS three times, and permeabilized and blocked with blocking solution (2% BSA, 0.1% Triton X-100 in PBS) for 20 min. Cells were incubated with anti-Flag (1:1000) (PM020, MBL) and anti-HA (1:1000) (M180-3, MBL) antibodies for 2 h. Cells were washed three times again and then incubated with anti-goat IgG-Alexa Fluor 568, anti-mouse IgG-Alexa Fluor 488, and anti-mouse IgG-Alexa Fluor 647 (Life Technologies); the nucleus were stained by DAPI. Images were taken using a Zeiss LSM-800 confocal microscope (Zeiss, Germany).

### Statistical analysis

Data are presented either from a representative experiment or as standard errors of the mean (SEM), from triplicate experiments. GraphPad Prism software (version 8) was used to make graphs (GraphPad Software).

## Results

### Identification of cellular proteins binding to the mutated HCV NS5B

**Fig. 1:**
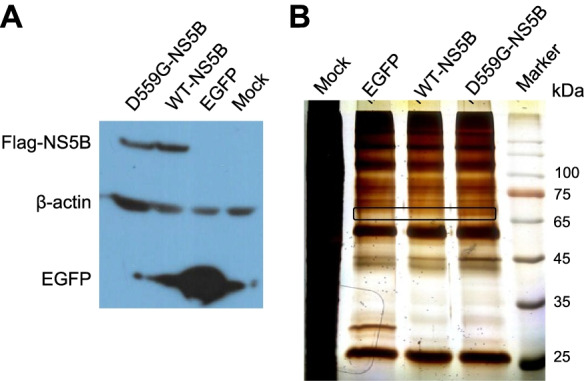
Ectopic expression and pull-down assay of D559G-NS5B and wild-type (WT)-NS5B. **A** Plasmids expressing Flag-tagged WT-NS5B and D559G-NS5B were transfected into 293T cells for 24 h, and NS5B expression was detected by western blot using anti-Flag antibody. Plasmid expressing EGFP was used as transfection control, and β-actin was detected as an internal control. **B** Silver staining of PAGE after separation of Flag-tagged NS5B-pull down mixtures. A band was visualized with more intensity in D559G-NS5B pellet (boxed), which was sliced and sent for mass spectrometry analysis, together with the gel slice from same position of WT-NS5B lane

We previously identified the NS5B mutation D559G (D2979G in polyprotein, the “G” of LSG mutations) critical for the initiation of HCV replication and virus production [5, 6]. Here, we hypothesized that presence of D559G may have changed the interaction of NS5B with host proteins, thus facilitating HCV replication. To test this hypothesis, we constructed NS5B wild-type (WT) and mutant D559G-NS5B, with Flag tag at N-terminus (n-Flag) and confirmed its expression in 293T cells (Fig. 1A). Since human hepatoma Huh7.5 cells are highly permissive HCV infection and has been widely used for HCV study, the pull-down experiments of WT-NS5B and D559G-NS5B were performed using transfected Huh7.5 cells (Fig. 1B). After silver staining of PAGE-separated pull-down proteins, an extra protein band was visualized in the D559G-NS5B pellet mixture (Fig. 1B). This band was sliced and sent for mass spectrometry analysis, together with the gel slice at same position of WT-NS5B pellet control (Fig. 1B). A number of proteins were identified specifically in D559G-NS5B pull-down pellet (Table 1). We decided to explore serine/threonine protein phosphatase 2A regulatory subunit delta isoform (PPP2R5D) in the HCV life cycle, as it was hit only in D559G-NS5B pull-down and was previously reported to be involved in the development and regulation of HCC as well as multiple diseases and pathways [14–16].

**Table 1:** Proteins discovered by mass spectrometry from HCV WT- and D559G-NS5B pull-down pellets

Proteins identified in mass spectrometry	D559G-NS5B pull-down	WT-NS5B pull-down
Immunoglobulin kappa variable 2D-26	+	+
Dermcidin	+	+
Phosphoglycerate kinase 1	+	+
Synaptopodin 2-like protein	+	+
Protein Shroom3	+	+
Hemoglobin subunit alpha	+	−
Hemoglobin subunit beta	+	−
L-2-hydroxyglutarate dehydrogenase, mitochondrial	+	−
Serine/threonine-protein phosphatase 2A 56 kDa regulatory subunit delta isoform (PPP2R5D)	+	−
Cartilage matrix protein	+	−
Kelch-like protein 25	+	−
Ribosomal protein S6 kinase beta-1	+	−
Putative coiled-coil domain-containing protein 144C	+	−
Transitional endoplasmic reticulum ATPase	+	−
Tensin-1	+	−
Probable G-protein coupled receptor 179	+	−
Hemicentin-1	+	−

The pull-down was performed using Huh7.5 cell lysates transfected with plasmids expressing D559G-NS5B or WT-NS5B. A protein band with high intensity in D559G-NS5B pull-down pellet was analyzed by mass spectrometry, and the gel slice at same position of WT-NS5B pull down was analyzed in parallel (Fig. 1B). Proteins that were found in D559G-NS5B band or both are presented. “ + ”, proteins were hit in the mass spectrometry; “−”, proteins were not hit in the mass spectrometry

### PPP2R5D interacted with NS5B

**Fig. 2:**
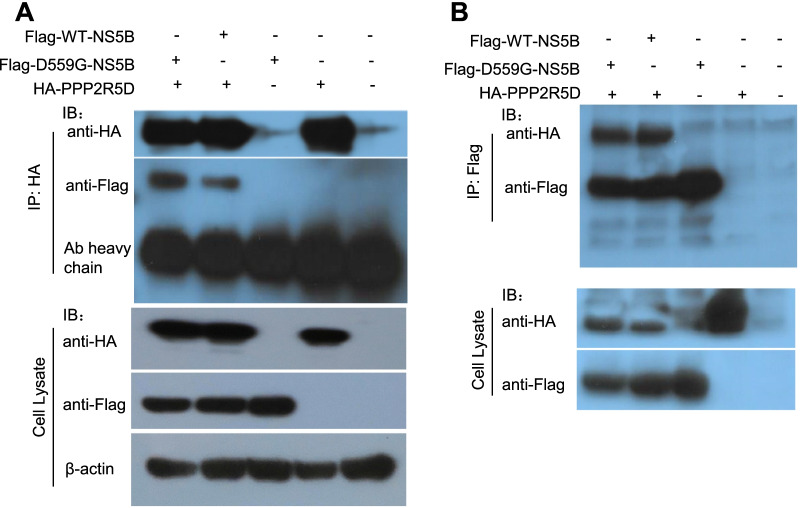
Co-IP of PPP2R5D with HCV WT-NS5B and D559G-NS5B. 293T cells were co-transfected with HA-tagged PPP2R5D and Flag-tagged WT-NS5B or D559G-NS5B, and the cells were lysed 48 h post-transfection and subjected to immunoprecipitation experiment. (A) Co-IP was performed using anti-HA antibody and immunoblotted (IB) with anti-HA or anti-Flag antibodies. The amount of D559G-NS5B pulled down by PPP2R5D was slightly more than that of WT-NS5B. (B) Co-IP was performed using anti-Flag antibody and immunoblotted with anti-HA or anti-Flag antibodies

To confirm that PPP2R5D interacted with HCV NS5B as seen in the pull-down assay, we performed co-immunoprecipitation (Co-IP) for HA-tagged PPP2R5D and Flag-tagged D559G-NS5B, in comparison with Co-IP for PPP2R5D and WT-NS5B. The Co-IP experiment was performed with either anti-Flag or anti-HA antibodies, and the results clearly showed that PPP2R5D interacted with D559G-NS5B and WT-NS5B (Fig. 2A and B). The amount of D559G-NS5B pulled down by PPP2R5D was slightly more than WT-NS5B (Fig. 2A), which may explain that PPP2R5D was hit only in D559G-NS5B pellet mixture (Fig. 1B).

**Fig. 3:**
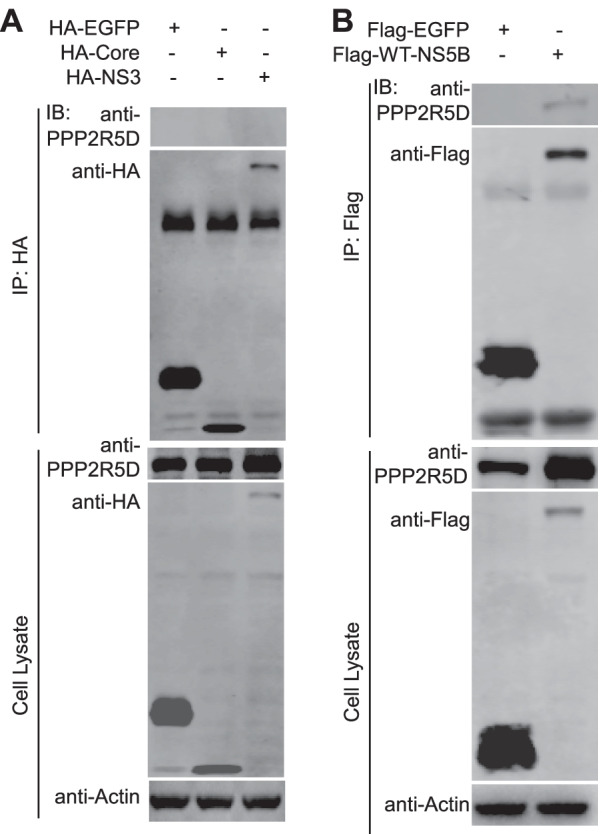
PPP2R5D specifically interacted with HCV NS5B. 293T cells were co-transfected with PPP2R5D and HCV Core, NS3, or NS5B for 36 h, and then the cells were lysed and subjected to Co-IP analysis. **A** Co-IP of Flag-PPP2R5D and HA-Core or HA-NS3; **B** Co-IP of HA-PPP2R5D and Flag-WT-NS5B, and Flag-EGFP was included as irrelevant IP control

To explore whether PPP2R5D specifically interacted with NS5B, we performed Co-IP experiment for PPP2R5D in the presence of other HCV proteins. We tested HCV Core and NS3 proteins, both being critical for HCV assembly and replication [17, 18]. We co-transfected Huh7.5 cells with Flag-PPP2R5D and HA-Core or Flag-PPP2R5D and HA-NS3 and performed Co-IP experiment. The results showed that PPP2R5D did not interact with Core and NS3 (Fig. 3A), as well as EGFP control, while expectedly PPP2R5D interacted with NS5B (Fig. 3B). Together, these results demonstrate that PPP2R5D specifically interacted with HCV NS5B.

### PPP2R5D colocalized with HCV NS5B in the ER

**Fig. 4:**
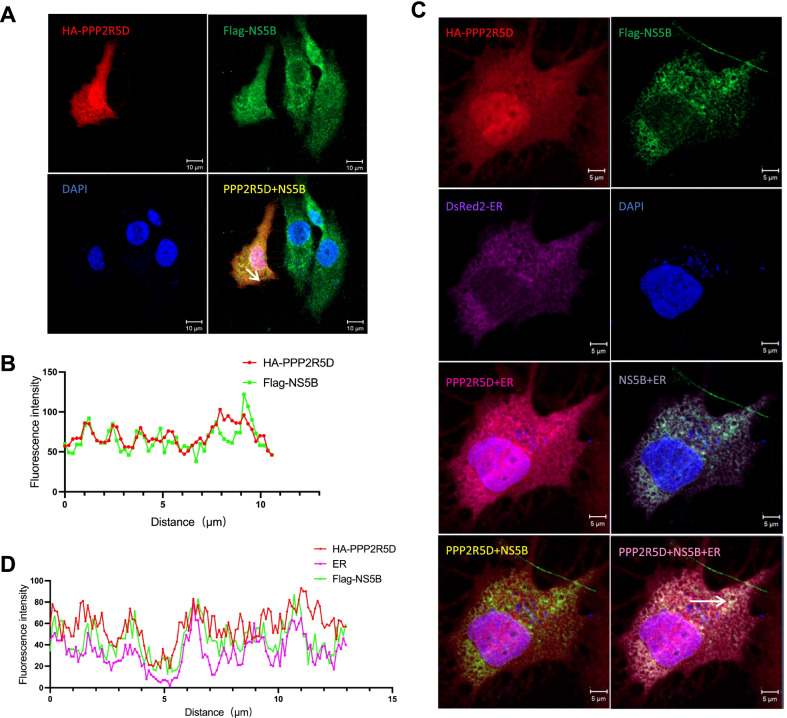
PPP2R5D and NS5B showed colocalization in the ER of Huh7.5 cells. Localization of PPP2R5D, NS5B, and ER were analyzed by confocal microscopy. **A** Huh7.5 cells were transfected with plasmids expressing HA-PPP2R5D and Flag-NS5B for 36 h, and then the cells were fixed and immunostained with anti-HA and anti-Flag primary antibodies plus corresponding secondary antibodies using channels 568 nm (indicated by red color) and 488 nm (green), respectively. The nucleus was stained by DAPI. Scale bar, 10 µm. **B** The colocalization of Flag-PPP2R5D and HA-NS5B was measured by fluorescence intensity line measurement (indicated by an arrow in the merged image in panel ***A***). **C** Huh7.5 cells were transfected with plasmids expressing HA-PPP2R5D, Flag-NS5B, and DsRed2-ER for 36 h, and then the cells were immunostained and visualized using channels 647 nm (HA-PPP2R5D, red), 488 nm (Flag-NS5B, green), and 568 nm (DsRed2-ER, purple). Scale bar, 5 µm. **D** The colocalization of Flag-PPP2R5D, HA-NS5B, and ER was measured by fluorescence intensity line measurement (the arrow in the merged image in panel ***C***)

Since PPP2R5D interacted with HCV NS5B, we set out to study whether PPP2R5D colocalizes with NS5B in the cell. We co-transfected Huh7.5 cells with Flag-NS5B and HA-PPP2R5D and monitored the localization of each protein by immune-confocal microscopy. After transfection, the cells positive for both proteins were analyzed for localization of each protein. The results revealed that PPP2R5D largely colocalized with NS5B (Fig. 4A). As PPP2R5D was previously found to localize in nucleus and cytoplasm [19, 20], and HCV NS5B was primarily localized in ER and membranous system [21–23], we further examined whether PPP2R5D affected the ER localization of NS5B. We transfected Huh7.5 cells with Flag-NS5B and HA-PPP2R5D singly or in combination, together with ER localizing reporter DeRed2-ER-5. It should be noted that somehow Huh7.5 cells were sensitive to the co-transfection of these three plasmids, as a number of cells showed morphological change after transfection, thus we performed localization analysis using the cells with a better shape. The results showed that PPP2R5D was localized to nucleus and cytoplasm (Fig. 4B), in line with previous observations [19, 20];  NS5B was primarily localized to ER and cytoplasm (Fig. 4B), which was also in agreement with the localization of NS5B found previously [21–23]. Colocalization analysis revealed that PPP2R5D and NS5B had colocalization signal in the ER (Fig. 4B and C), and no apparent difference of ER-localized NS5B was observed in the presence of PPP2R5D. Together, these data show that the distribution of HCV NS5B were not distinguishably changed by PPP2R5D, and PPP2R5D had colocalization with NS5B in the ER.

### PPP2R5D was required for HCV infection in cultured cells

**Fig. 5:**
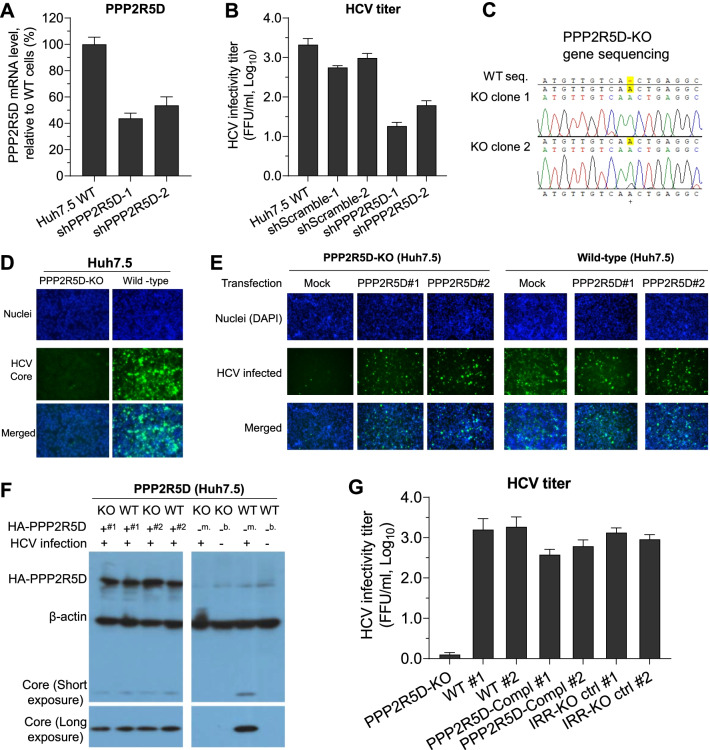
PPP2R5D was required for HCV infection in Huh7.5 cells demonstrated by gene knockdown, knockout, and stable complementation. **A** shRNAs targeting PPP2R5D (shPPP2R5D-1 and -2) suppressed PPP2R5D mRNA expression in Huh7.5 cells. The mRNA level of PPP2R5D in the shRNA-treated Huh7.5 cells was determined by real-time RT-PCR using GAPDH as internal control. Data are the mean of three determinations with the standard error of the mean (SEM). **B** HCV infectivity titers (FFU/ml, log_10_) were decreased in shPPP2R5D-knockdown Huh7.5 cells, in comparison with non-treated WT and control shRNAs (shScramble-1 and -2)-treated Huh7.5 cells. Culture supernatant was collected on day 7 post infection, and HCV infectivity titers were determined by FFU assay. Data are the mean of three determinations plus SEM. **C** PPP2R5D disruption in puromycin-selected monoclonal Huh7.5 cell line. Monoclonal PPP2R5D-KO cell line was sequenced, and one nucleotide insertion was found in PPP2R5D gene. **D** HCV infection was dramatically decreased in PPP2R5D-KO cells, compared to WT Huh7.5 cells at day 7. **E** Transient expression of PPP2R5D restored HCV infection in PPP2R5D-KO cells. The wild-type (WT) and PPP2R5D-KO Huh7.5 cells were transfected with HA-tagged PPP2R5D for 24 h (“#1” and “#2” represent two transfections), HCV infection was performed with MOI of 0.01, and immunostaining of HCV Core was performed at 48 h post infection. **F** Western blot analysis of PPP2R5D expression and HCV Core in transfected WT and PPP2R5D-KO Huh7.5 cells. The cells were collected from the experiment shown in panel ***E***. “#1” and “#2” indicate the cells from duplicate transfections; “m.” indicates mock transfection; “b.” indicates the cells non-transfected (blank control). **G** Stable complementation of PPP2R5D in PPP2R5D-KO cells (PPP2R5D-Compl) rescued HCV infection. The WT, PPP2R5D-Compl, and irrelative knockout control cells (IRR-KO) Huh7.5 cells were infected with HCV (MOI = 0.01) in duplicate (#1 and #2), and the culture supernatant was collected at day 5 and determined for the infectivity titers by FFU assay. The means of three determinations plus FFU are shown

As PPP2R5D interacted with HCV NS5B, we proceeded to investigate whether PPP2R5D is required for HCV infection. Initially, we constructed two lentivirus-based shRNAs for PPP2R5D-knockdown (PPP2R5D-KD), and both shPPP2R5D-1 and -2 could decrease PPP2R5D mRNA levels by 50%-60% in Huh7.5 cells (Fig. 5A). Then, we infected WT and PPP2R5D-KD Huh7.5 cells with genotype 2a recombinant J6^5’UTR−NS2^/JFH1 [12] and found that HCV infectivity titers released by PPP2R5D-KD cells was lower by ~ 55-fold than that by scramble shRNAs-treated or WT Huh7.5 cells (Fig. 5B). These results suggest that PPP2R5D supported HCV infection of Huh7.5 cells.

To further validate the supportive role of PPP2R5D in HCV infection, we generated PPP2R5D-knockout Huh7.5 cell lines (PPP2R5D-KO) using CRISPR/Cas9 technology. PPP2R5D-KO cell clones were confirmed by DNA sequencing, and one nucleotide insertion was found in the PPP2R5D gene, thus creating a frameshift mutation of PPP2R5D (Fig. 5C). Infection experiment revealed that PPP2R5D-KO cells were hardly infected by HCV at day 7 post infection, when compared with WT Huh7.5 cells (Fig. 5D). To examine whether complementation of PPP2R5D rescues HCV infection in PPP2R5D-KO cells, we transfected PPP2R5D-KO and WT Huh7.5 cells with HA-tagged PPP2R5D. The results showed that transfection of PPP2R5D restored HCV infection in PPP2R5D-KO cells to a level close to that in WT cells; expectedly, PPP2R5D-KO cells of mock transfection showed a very low level of HCV infection (Fig. 5E). Transfection of PPP2R5D in WT Huh7.5 cells did not increase HCV infection, but resulted in a slight reduction of HCV infection, which might suggest that endogenous PPP2R5D is sufficient for HCV infection and the plasmid-transfection procedure slightly affected the efficiency of HCV infection (Fig. 5E); meanwhile, the expression of PPP2R5D and HCV Core protein of transfected cells were confirmed by western blotting (Fig. 5F).

Next, we constructed an untagged PPP2R5D-expressingg plasmid and transfected into PPP2R5D-KO cells to generate a PPP2R5D-KO cell line stably expressing PPP2R5D and designated PPP2R5D-Compl cell line. The PPP2R5D-Compl cells supported HCV infection and released HCV infectivity titers close to WT Huh7.5 cells (Fig. 5G). Taken together, these results demonstrate that PPP2R5D was required for HCV infection in cultured cells.

### PPP2R5D promoted HCV RNA replication

**Fig. 6:**
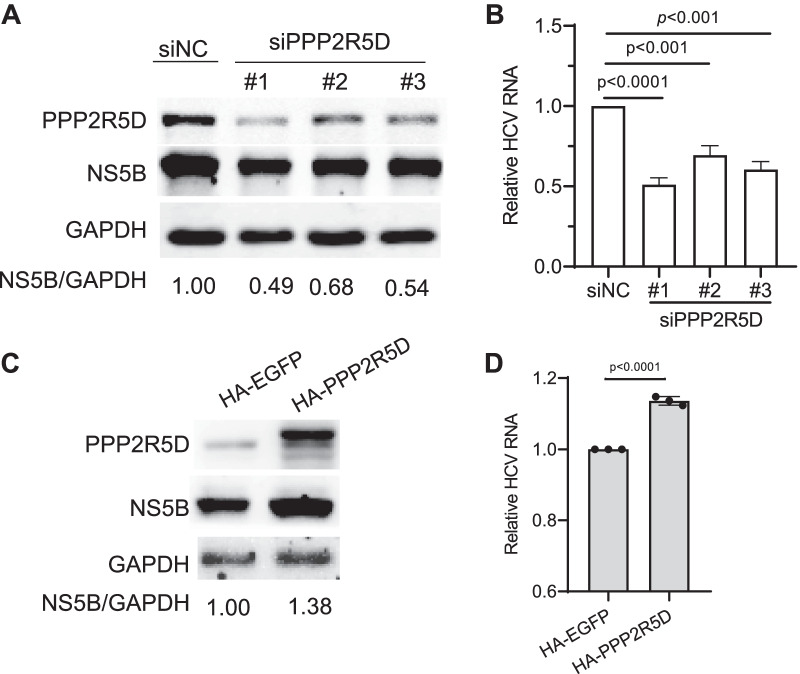
PPP2R5D enhanced HCV replication. (**A** and **B**) siRNA silencing of PPP2R5D decreased HCV replication in replicon-harboring cells. HCV genotype 2a replicon JFH1-SGRep replicon expressing cells were generated and used for silencing experiment. siRNAs targeting different regions of PPP2R5D (#1, #2, and #3) were transfected into the replicon cells for 36 h. The expression of NS5B protein level was detected by western blot and normalized to GAPDH (panel ***A***), and viral RNA levels were determined using qRT-PCR and relative to that in the control siRNA-treated cells (panel *B*). (**C** and **D**) Over-expression of PPP2R5D increased HCV replication in replicon cells. HA-PPP2R5D was transfected into HCV replicon cells for 36 h, HA-EGFP being a transfection control, and then HCV replication was evaluated for NS5B protein by western blot and normalized to GAPDH (panel ***C***), and viral RNA levels were determined and relative to that of EGFP-transfected control cells (panel **D**). Images of western blot are representative of three independent experiments, while the data of viral RNAs was mean value with SEM of three independent experiments

Since PPP2R5D was required for HCV infection, we furthered to investigate the underlying mechanism by which PPP2R5D facilitated HCV infection. We examined whether PPP2R5D affect HCV replication using HCV replicon system. The Huh7.5 cells stably harboring genotype 2a JFH1 subgenomic replicon was generated and used, as previously described [24]. Transfection of three PPP2R5D-spefic siRNAs (siPPP2R5D #1, #2, or #3) individually knocked down PPP2R5D in replicon cells (Fig. 6A). Concomitantly, both HCV NS5B protein (Fig. 6A) and viral RNA (Fig. 6B) were decreased in PPP2R5D-knockdown replicon cells compared to siNC-treated control cells. In addition, we also transfected HA-tagged PPP2R5D into JFH1 replicon cells and found that PPP2R5D increased HCV replication, showing an increased NS5B protein and viral RNA levels (Fig. 6C and D). Tougher, these data demonstrate that PPP2R5D facilitated HCV RNA replication.

### The phosphatase activity of PP2A was not affected by HCV NS5B

**Fig. 7:**
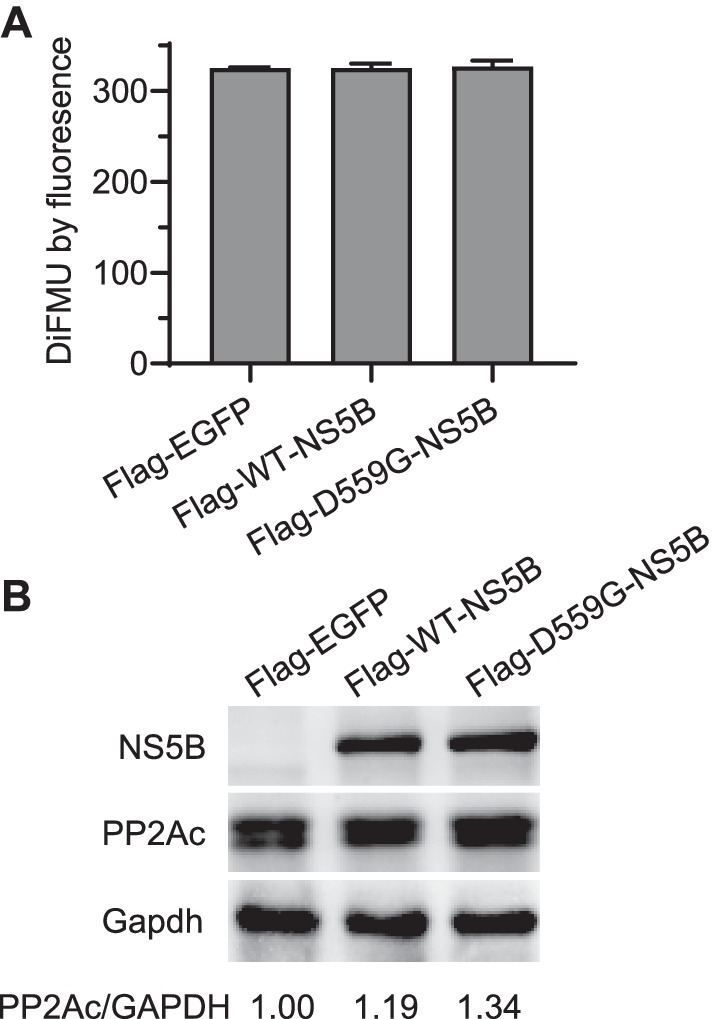
The phosphatase activity of PP2A was not affected by HCV NS5B. **A** Huh7.5 cells were transfected with Flag-WT-NS5B, Flag-D559G-NS5B, or control EGFP for 36 h, and then the cells were lysed and subjected for phosphatase activity assay. *Y* axis shows the absolute value of DiFMU present in the solution determined by fluorescence reads. **B** The core enzyme catalytic subunit C (PP2Ac, alpha and beta isoforms) was not changed by HCV NS5B. Huh7.5 cells were transfected with Flag-WT-NS5B, Flag-D559G-NS5B, or control EGFP for 36 h, and then the cells were lysed and analyzed by western blot; the levels of PP2Ac (α and β isoforms) were normalized to GAPDH

As PPP2R5D is an isoform of the subunit family B56 of the enzyme serine/threonine-protein phosphatase 2A (PP2A) involved in HCC, multiple diseases, and cellular pathways [15, 16], we proceeded to investigate whether the interaction and colocalization between PPP2R5D and HCV NS5B affected the activity of PP2A. To this end, we transfected Huh7.5 cells with Flag-tagged WT-NS5B and D559G-NS5B for 36 h, and the cells lysates were subjected to phosphatase measurement using DiFMUP assay [25]. The results showed that the phosphatase activity of PP2A was not changed by either WT-NS5B or D559G-NS5B (Fig. 7A). To further examine whether NS5B affected the core enzyme subunit of PP2A, the catalytic subunit C (alpha and beta isoforms), we analyzed the protein level of each subunit after transfection of HCV NS5B. The results showed that the levels of subunit C (alpha and beta isoforms) were not apparently changed by HCV NS5B transfection (Fig. 7B). Together, expresion of HCV NS5B did not apparently affect the phosphatase activity of PP2A.

## Discussion

In this study, we identified PPP2R5D as a cellular factor required HCV infection in Huh7.5 cells. PPP2R5D was first identified in NS5B pull-down assay and further studied its role in HCV infection through PPP2R5D knockout, complementation, virus infection, and replicon analysis. We provided strong evidences that knockout of PPP2R5D eliminated HCV infection in Huh7.5 cells, while transient and stable complementation of PPP2R5D rescued HCV infection. Mechanistic study uncovered that PPP2R5D affected HCV replication, but its phosphatase activity was not apparently affected by NS5B. In addition, interaction and ER colocalization of PPP2R5D and HCV NS5B were demonstrated by Co-IP and confocal microscopy, respectively. Together, this study provides strong evidences that PPP2R5D served as a cellular factor necessary for HCV infection, thus facilitating future investigation on the functional role of PPP2R5D in the HCV life cycle and viral pathogenesis.

PPP2R5D is a subunit of PP2A enzymes, which is involved in many regulating pathways and turns on or off gene expression by removing the phosphate group from proteins [16]. Protein phosphatase 2A (PP2A) is a major and multifunctional serine/threonine-specific phosphatase consist of structural subunit A, a regulatory subunit B, and core enzyme catalytic subunit C [26]. PPP2R5D plays a major role in negative regulation of the PI3K/AKT signaling pathway, autism or other brain related disorders [26]. PP2A have been evaluated in cancer progression and these group of proteins have shown the interaction with different HCV proteins [16, 27–29]. A recent study demonstrated that PPP2R5 phospho-regulators  were degraded by HIV Vif protein and affected HIV pathogenesis [30]. Development of HCC remains a major concern of long-term chronic HCV infection, and progression to liver cancer or related diseases was not ceased satisfactorily even in the patients achieving sustained virologic response after anti-HCV therapy. Recently, it was reported that mice lacking PPP2R5D spontaneously develop HCC [14]. Given the importance of PPP2R5D in regulating cell physiology, different diseases, and being a tumor-suppressive Ser/Thr protein phosphatase [31], it will be interesting to explore its role in HCV-related HCC. Although we have demonstrated the indispensable role of PPP2R5D in HCV infection of cultured cells through enhancing virus replication, the underlying mechanism of PPP2R5D in the HCV life cycle warrants further investigation.

## Conclusions

We have demonstrated that cellular PPP2R5D protein was required for HCV infection in hepatoma cells. PPP2R5D interacted and colocalized with HCV NS5B, which may contribute to its enhancement effect for HCV RNA replication. This study discovers a new host factor important for the HCV life cycle, thus contributing to the studies of virus-host interaction and the pathogenesis of HCV.

## Abbreviations

HCV: Hepatitis C virus
PPP2R5D: Serine/threonine protein phosphatase 2A regulatory subunit delta isoform
HCC: Hepatocellular carcinoma
DAAs: Direct-acting antivirals
UTRs: Untranslated regions
IRES: Internal ribosomal entry site
NS: Non-structural
WT-NS5B: Wild-type NS5B
FFU: Focus forming unit
DMEM: Dulbecco’s modified Eagle’s medium
shRNA: Short hairpin RNA
siRNA: Interfering RNA
GAPDH: Glyceraldehyde-3-phosphate dehydrogenase
SEM: Standard errors of the means
Co-IP: Co-immunoprecipitation
KD: Knockdown
KO: Knockout
